# Pubertal development in 46,XY patients with *NR5A1* mutations

**DOI:** 10.1007/s12020-021-02883-y

**Published:** 2021-10-06

**Authors:** Isabel Mönig, Julia Schneidewind, Trine H. Johannsen, Anders Juul, Ralf Werner, Ralf Lünstedt, Wiebke Birnbaum, Louise Marshall, Lutz Wünsch, Olaf Hiort

**Affiliations:** 1 Division of Paediatric Endocrinology and Diabetes, Department of Paediatric and Adolescent Medicine, University of Lübeck, Lübeck, Germany; 2 Department of Growth and Reproduction and International Center for Research and Research Training in Endocrine Disruption of Male Reproduction and Child Health (EDMaRC), Rigshospitalet, University of Copenhagen, Copenhagen, Denmark; 3 Institute for Molecular Medicine, University of Lübeck, Lübeck, Germany; 4 Catholic Children’s Hospital Wilhelmstift, Hamburg, Germany; 5 Department of Paediatric Surgery, University of Lübeck, Lübeck, Germany

**Keywords:** Differences of sex development, NR5A1 mutation, Pubertal development, Virilization

## Abstract

**Purpose:**

Mutations in the *NR5A1* gene, encoding the transcription factor Steroidogenic Factor-1, are associated with a highly variable genital phenotype in patients with 46,XY differences of sex development (DSD). Our objective was to analyse the pubertal development in 46,XY patients with *NR5A1* mutations by the evaluation of longitudinal clinical and hormonal data at pubertal age.

**Methods:**

We retrospectively studied a cohort of 10 46,XY patients with a verified *NR5A1* mutation and describe clinical features including the external and internal genitalia, testicular volumes, Tanner stages and serum concentrations of LH, FSH, testosterone, AMH, and inhibin B during pubertal transition.

**Results:**

Patients who first presented in early infancy due to ambiguous genitalia showed spontaneous virilization at pubertal age accompanied by a significant testosterone production despite the decreased gonadal volume. Patients with apparently female external genitalia at birth presented later in life at pubertal age either with signs of virilization and/or absence of female puberty. Testosterone levels were highly variable in this group. In all patients, gonadotropins were constantly in the upper reference range or elevated. Neither the extent of virilization at birth nor the presence of Müllerian structures reliably correlated with the degree of virilization during puberty.

**Conclusion:**

Patients with *NR5A1* mutations regardless of phenotype at birth may demonstrate considerable virilization at puberty. Therefore, it is important to consider sex assignment carefully and avoid irreversible procedures during infancy.

## Introduction

The term “Differences (or Disorders) of Sex Development” (DSD) summarizes mostly hereditary conditions with a discrepancy between a person’s chromosomal, gonadal, and phenotypic sex [1]. These conditions occur rarely with a prevalence of about 1 per 5000 live births [2]. Despite advances in genetic diagnostics, the underlying genetic cause in many of these patients remains elusive [3, 4].

One genetic cause for DSD, especially in individuals with 46,XY karyotype, is mutations in the *NR5A1* (*Nuclear receptor subfamily 5, group A, member 1*) gene. *NR5A1* encodes the transcription factor Steroidogenic Factor-1 (SF-1) that plays a pivotal role in adrenal and gonadal development as well as in steroidogenesis. SF-1 is expressed in the bipotential gonad and regulates its differentiation towards testes and ovaries [5]. In testes, SF-1 initiates the transcription of the *Sry-related HMG Box-9* (*SOX9)* gene, which eventually results in the differentiation of the precursor cells towards Sertoli cells [6–8]. Here, SF-1 activates the expression of the Anti-Müllerian hormone (AMH) and consequently, it influences the regression of the paramesonephric ducts [9, 10]. Besides, SF-1 is involved in virilization of the internal and external genitalia through the regulation of steroidogenesis in Leydig cells [11–13]. In ovaries, SF-1 is expressed in granulosa and theca cells and is as well involved in pathways of steroid synthesis [14].

In patients with 46,XY DSD, mutations in the *NR5A1* gene are associated with a broad phenotypic spectrum comprising isolated hypospadias [15–17], ambiguous external genitalia with a scrotum bipartitum and/or micropenis [18–20] up to completely female external genitalia [21–23]. Additionally, in some of these patients, Müllerian remnants might be detected [19, 24]. Furthermore, a specific *NR5A1* mutation (p.R92W) has recently been described that might leads to remarkable virilization in patients with 46,XX testicular DSD [25, 26]. This underlines the importance of SF-1 for differentiation of testes and ovaries and illustrates the broad phenotypic spectrum in patients with *NR5A1* mutations.

To date, little is known about pubertal development in patients with *NR5A1* gene mutations. Thus, the aim of this study was to characterize a cohort of 10 46,XY patients with genetically verified *NR5A1* mutations and varying phenotypes at birth and to describe their long-term follow-up during adolescence. Detailed clinical and hormonal data during pubertal development are provided.

## Methods

### DNA analyses

Genomic DNA was isolated from peripheral blood cells by standard procedures. The *NR5A1* exons 1-7 including all exon-intron boundaries were amplified and sequenced by direct cycle sequencing using the BigDye Terminator v1.1 Cycle Sequencing Kit (Applied Biosystems, USA) and the 3130 Genetic Analyser (Applied Biosystems, USA). Sequence analysis was conducted using SeqScape3 (Applied Biosystems, USA). Functional analysis of mutations p.Y211TfsX83, p.T40P and p.L230R as well as in silico analysis of p.T40P and p.L230R were conducted as described earlier [23, 24]. For the functional prediction of missense mutation p.V369F and in-frame deletion p.P221_L230del different prediction algorithms were used, i.e., PolyPhen-2, Mutation Taster, and SIFT.

### Hormone analyses/reference data

Reference ranges for LH and FSH were based on time-resolved fluoroimmunometric assays (AutoDELFIA, Perkin Elmer, Turku, Finland) as previously reported [27]. The reference range for testosterone was based on liquid chromatography-tandem mass spectrometry-methodology and is previously published [28, 29]. The reference range for AMH was based on a chemiluminescence immunoassay (Access 2, Beckman Coulter, Brea, CA, USA) after internal method comparison with a previously published reference range [30]. The reference range for inhibin B was based on an enzyme-linked immunosorbent assay (Beckman Coulter Inhibin B Gen II ELISA, Beckman Coulter, Brea, CA, USA) after internal method comparison and factorization of a previously published reference range [31]. Lower detection limits were 0.05 IU/L for LH and FSH, 0.10 nmol/L for testosterone, 2 pmol/L for AMH, and 3 pg/mL for inhibin B. The reference ranges were established at The Department of Growth and Reproduction, Rigshospitalet, Copenhagen, Denmark, using a Generalized Additive Model for Location, Scale and Shape (GAMLSS) as previously described [29]. All hormone analyses were accredited according to DS/EN ISO 15189 by The Danish Accreditation Fund. Hormonal values of the presented patients were evaluated through different methods by various laboratories. The results were secondly converted into standard units to be comparable with the reference data.

### Testicular volume/ Reference data

Testicular volume was measured by clinical palpation. Standard deviations scores were calculated according to Joustra SD et al. [32]. Reference data for testicular volume were based on reference charts for testicular volume in Dutch children and adolescents [32].

### Patients

**Fig. 1:**
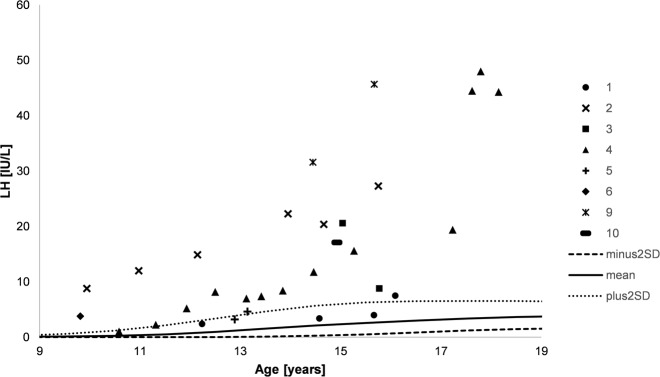
LH levels during course of puberty. Numbers indicate the different patients, lines indicate plus and minus two standard deviations as well as mean (male reference data)

**Fig. 2:**
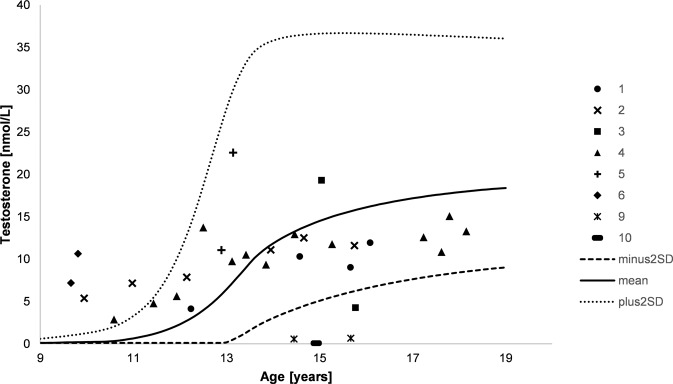
Testosterone levels during course of puberty. Numbers indicate the different patients, lines indicate plus and minus two standard deviations as well as mean (male reference data)

**Fig. 3:**
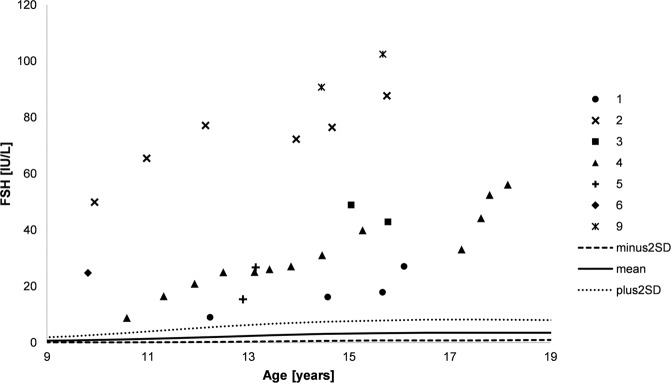
FSH levels during course of puberty. Numbers indicate the different patients, lines indicate plus and minus two standard deviations as well as mean (male reference data)

**Fig. 4:**
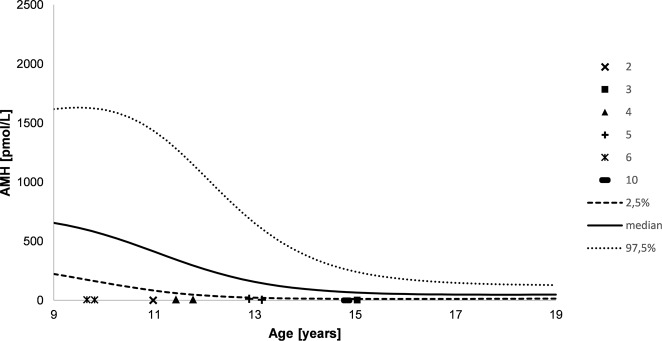
AMH levels during course of puberty. Numbers indicate the different patients, lines indicate plus and minus two standard deviations as well as median (male reference data)

**Fig. 5:**
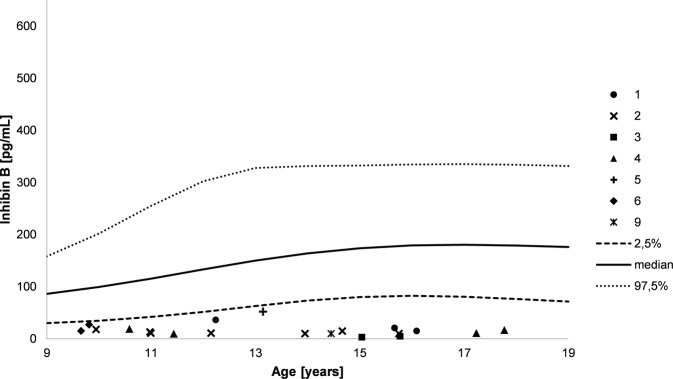
Inhibin B levels during course of puberty. Numbers indicate the different patients, lines indicate plus and minus two standard deviations as well as median (male reference data)

**Fig. 6:**
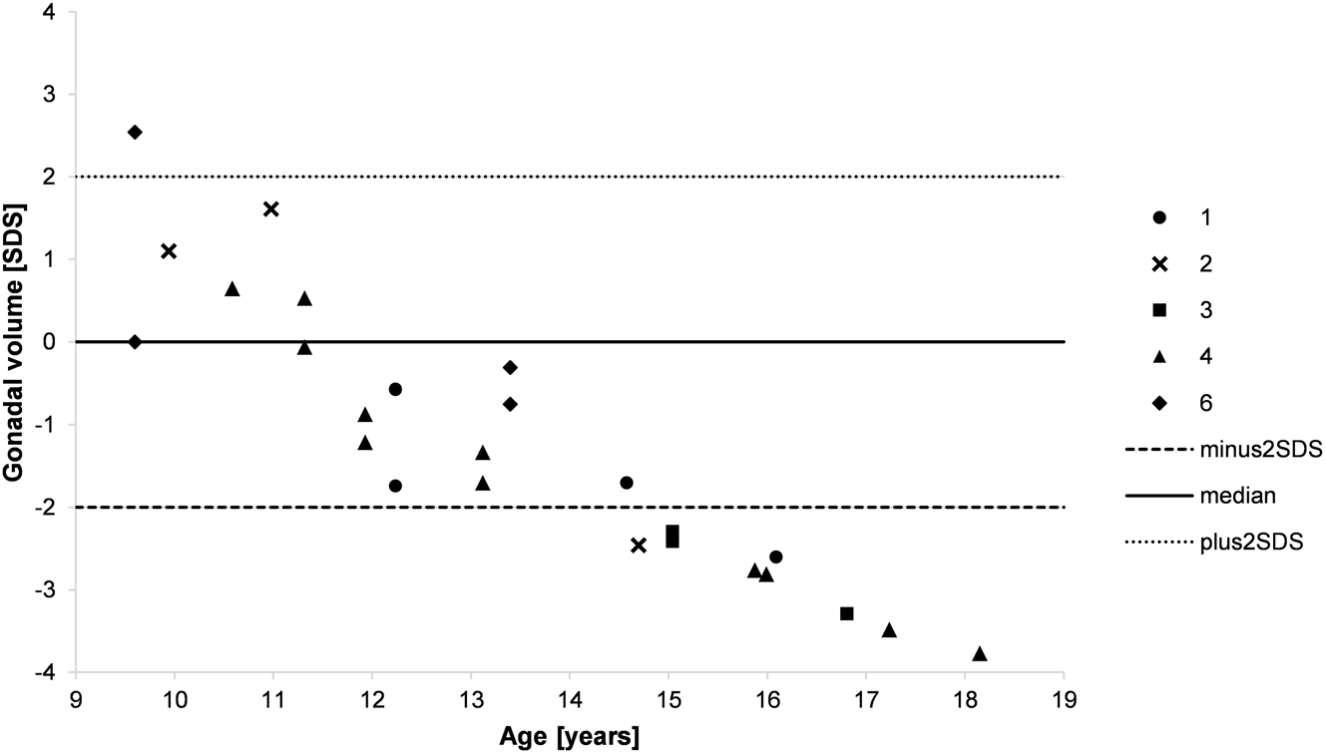
Standard deviation of palpable gonadal volume. Numbers indicate the different patients, lines indicate plus and minus two standard deviations as well as median. Reference data were calculated according to [32]. SDS *=* Standard deviation score

All DSD patients in or beyond pubertal age where a *NR5A1* mutation had been detected at the laboratory of the University of Lübeck and sufficient available clinical data were included in this study. Patients were either directly treated at the University Hospital of Lübeck or DNA and clinical information was transferred by their primary endocrinologist for further evaluation. All patients gave their written informed consent to genetic analysis of the *NR5A1* gene for scientific purposes and publishing their anonymized data. The study was approved by the Ethical Committee of the University Hospital of Lübeck, Lübeck, Germany (AZ: 08–081). Clinical and genetic findings are indicated in Table 1 and Fig. 6. Hormonal values are shown in Figs. 1–5.

**Table 1:** Clinical and genetic data of patients

Patient	*NR5A1* mutation/ Age at karyotype analysis/ genetic diagnosis	Age at first evaluation	Clinical signs/ External genitalia at first evaluation/ Hormonal data in first six months of life	Signs of virilization at pubertal age/ Gonadal volume (right/left)	Location of gonads (right/left)	Müllerian structures/Detection method	Gender	Hormone therapy/Surgery [age]	Additional information
**1**	c.630_636del p.Y211TfsX83 4.5 months/ 12.8 y	1 day	Scrotal hypospadias Phallic length 2.4 cm Bifid scrotum EGS 3.5 1 day: T 6.5 nmol/l; 4.3 months: T 3.3 nmol/l, LH 2.6 IU/l, FSH 3.7 IU/l	12.2 y: Tanner stage G3/PH1/ GV 2/4 ml 14.6 y: Tanner stage G4/PH4/ GV 7/7 ml 16.1 y: Tanner stage G4/PH4/ GV 8/8 ml	ing/ing	no/ultrasound	male	No Male genital reconstructive surgery [infant]	Sibling of patient 6; previously published in [24]
**2**	c.1200–1201del p.L401AfsX2 approx. 7 days/ 12.2 y	1 day	Penoscrotal hypospadias Phallic length 1.0 cm Rugated labioscrotal folds EGS 4.5 4.0 months: T 0.1 nmol/l, FSH 19.5 IU/l	9.9 y: Tanner stage G3/PH4/A2, beginning pubertal vocal change/ GV 3.5/3.5 ml 14.7 y: Tanner stage G4/PH4/ GV 2.5/2.5 ml	scr-ing/scr	no/MRI	female, change to male at 1 week	No hormone therapy No surgery	
**3**	c.312–317 delinsAGAAGAAGGC p.L105EfsX45 10 days/ 15.0 y	10 days	Penoscrotal hypospadias Phallic length 1.0 cm Bifid scrotum EGS 3.5 10 days: T 2.2 nmol/l, LH 4.2 IU/l, FSH 3.7 IU/l	15.0 y: Tanner stage PH5, phallic length 3–4 cm, diameter 1.5–2 cm/ GV 4/5 ml	ing/ing	no/ultrasound	female, change to male at 6 months	Since 15.8 y: Testosterone Male genital reconstructive surgery [infant]	
**4**	c.118 A > C p.T40P 12 days/ 15.0 y	26 days	Proximal hypospadias Phallic length 1.3 cm Prominent labioscrotal folds EGS 5.5 1.1 months: T 0.7 nmol/l, LH 3.9 IU/l, FSH 13.2 IU/l	10.6 y: Tanner stage G2-3/ GV 3.5/3.5 ml 11.3 y: Tanner stage G2-3/PH/ GV 4.5/3.5 ml 13.1 y: Tanner stage G4/PH4/ GV 4/3 ml 16.5 y: Tanner stage G4/PH5/ GV 5/5 ml 17.2 y: Tanner stage PH5, hypoplastic phallus/ 4.5/4.5 ml 18.2 y: phallic length 8 cm, PH4/ GV 3.5/3.5 ml	scr/scr	no/laparoscopy	female, change to male at 2 months	15.2–16.7 y: Testosterone 16.7–17.4 y: Dihydrotestosterone 17.4–17.7 y: Choriongonadotropine Male genital reconstructive surgery [infant]	Spermiogram at 17.1 y: azoospermia; previously published in [24]
**5**	c.1361–1377dup p.Q460KfsX42 approx. 10 months/ 13.1 y	11 months	Clitoromegaly Rugated labioscrotal folds	12.5 y: increase of clitoral length, growth of pubic hair	scr/scr	no/ultrasound	female	Since 13.1 y: GnRH analogue No surgery	
**6**	c.1005 G > T p.V359F 9.8 y/ 13.4 y	9.6 y	Penoscrotal hypospadias Bifid scrotum Tanner stage G2-3/ PH5/A1	9.6 y: Tanner stage G2-3/PH5/ GV 5.5 ml/NA 13.4 y: Tanner stage G3/PH5/ GV 9/7 ml	scr/ing	no/MRI	female, change to male at 9 years	No hormone therapy Male genital reconstructive surgery [early puberty]	9.8 y: accelerated skeletal age
**7**	c.662–691del p.P221_L230del approx. 13 y/ 31.5 y	13.1 y	No menarche Tanner stage B1/PH1	15 y: increase of clitoral length (2-3 cm)	NA	yes/MRI	female	16–29 y: Estradiol Since 29 y: Estradiol+ Gestagen/Gonadectomy, Clitoral reduction [17 y]	
**8**	c.689 T > G + polymorphism c.437 G > C p.L230R + polymorphism p.G146A NA/ 18.2 y	14.3 y	No menarche Clitoromegaly Tanner stage B1/A2/PH6	14 y: increase of clitoral length, growth of axillary and pubic hair	ing/ing	no/laparoscopy	female	14.3–17.8 y: GnRH analogue Since 14.3 y: Conjugated estrogens Gonadectomy [17.8 y]	At pubertal age: Hirsutism Lipomastia Obesity (BMI 31 kg/m^2^); previously published in [23]
**9**	c.630_636del p.Y211TfsX83 14.5 y/ 15.0 y	14.5 y	No menarche Prominent clitoris Tanner stage B1/PH1	14 y: No signs of virilization	abd/abd	yes/laparoscopy	female	Since 15.7 y: Estradiol Gonadectomy [15.7 y]	Sibling of patient 1; previously published in [24]
**10**	c.522_534del insGGGCCACTGGCTGGCTA p.A176HfsX22 14.9 y/ 17.0 y	14.8 y	No menarche Clitoromegaly Sinus urogenitalis Tanner stage B3/PH4-5	13 y: increase of clitoral length, breast development, weight gain, deepening voice	abd/ing	yes/MRI	female	14.9–15.3 y: GnRH analogue 15.0–15.6 y: Estradiol 15.6–18.2 y: Estradiol+Gestagen 18.2 y–18.9 y: Estradiol patch Since 18.9 y: Estradiol Gonadectomy [15.3 y]	

*T* Testosterone, *LH* Luteinizing hormone, *FSH* Follicle Stimulating Hormone, *GV* Gonadal Volume, *src* Scrotal, *ing* Inguinal, *abd* Abdominal, *y* Years, *NA* Not Available, *Approx.* Approximately.

## Results

Here we describe the long-term follow-up of 10 individuals from nine separate families with a typical 46,XY karyotype and different heterozygous mutations in the *NR5A1* gene (Table 1). Median follow-up time of patients was 11.0 years (range 1.5–19.3 years).

### Age at first evaluation

Patients 1–5 presented and underwent first evaluation during infancy at a median age of 2.5 months (range 1 day–11 months). In patient 6, ambiguous genitalia were noticed at birth, but the first detailed medical evaluation was conducted at the age of around 9 years due to increased signs of virilization. In contrast, patients 7–10 first became symptomatic at a median age of 14.2 years (range 13.1 years–14.8 years) and were first evaluated at the age of puberty.

### Presenting symptoms

In patients 1–4, ambiguous genitalia with signs of undervirilization and significantly reduced external genitalia scores (EGS; normal > 10.5 [33]) were noticed at birth with a median EGS of 4.3 (range 3.5–5.5). Patient 5 showed a clitoromegaly and rugated labioscrotal folds at birth but EGS in this patient as well as in patient 6 could not be calculated due to insufficient phenotypic information.

In patients 7–10, no signs of ambiguous genitalia were reported at birth but according to the patients or their parents they had apparently female external genitalia. These patients presented first at the age of puberty. Main reasons for presentation of patients 5–10 at pubertal age were either signs of virilization and/or pubertal delay with absent thelarche and/or menarche.

### Internal genitalia

In patients 1–6, inguinal or scrotal gonads were palpable, no one showed abdominal located gonads. In patients 7–10, the gonads were located labioscrotal, inguinal, and/or abdominal.

Furthermore, in seven patients, Müllerian structures could not be detected by ultrasound or MRI, respectively. By contrast, a uterus was found in three patients (patients 7, 9, and 10).

### Clinical course of puberty

Presentation at puberty occurred later in patients who lived as females at that time (median age 13.1 years, range 9.6–14.8 years) than in patients who lived as males (median age 11.9 years, range 9.9–15.0 years).

Patients who lived as males at time of puberty (patients 1–4 and 6) showed signs of spontaneous puberty with increasing virilization like growth of phallic length or development of axillary and pubic hair. However, in all cases palpable gonadal volume stayed below average or even decreased over time of puberty (patient 2 and 4, Fig. 6). Pubertal progression was reflected by the increase of Tanner stages (Table 1). There was no evidence for pubertal delay in individuals who lived as males during puberty. In contrast, patients 2, 4, and 6 showed rather early pubertal development.

Patients who lived as females at time of puberty showed variable symptoms. Patients 5, 8, and 10 presented for evaluation because of an increase in clitoral length as well as progression of pubertal hair growth and/or absent thelarche and/or menarche (patients 7–10) at the age of 12.5 years until 14.8 years. Patient 7 presented with an enlarged clitoris but without development of axillary or pubic hair. Patient 9 was the only patient without signs of virilization at pubertal age. Patient 10 showed spontaneous breast development with a Tanner stage of B3 at the age of 14.8 years.

### Hormonal data during puberty

The hormonal values of all studied patients during puberty are shown according to sex- and age-related reference ranges in Figs. 1–5.

In seven patients with available hormonal data (patients 2–4, 6, and 8–10), serum concentrations of LH were above the male reference range in all measurements and further increased during puberty (Fig. 1). Patient 1 and 5 had LH levels within the male reference range until the age of 15.7 years (ref. range 0.49–6.26 IU/L) and 12.9 years (ref. range 0.02–3.36 IU/L), respectively, that increased above the male reference range in the further course.

Interestingly, serum concentrations of testosterone were elevated during early puberty in three patients from the age of 9.7 to 11.0 years (patients 2, 4, and 6, Fig. 2). In seven patients (patients 1–6 and 8), testosterone concentrations were within the male reference range in the further course. In one of these patients (patient 3), testosterone declined below the reference range at the age of 15.8 years. In patients 9 and 10, a hypergonadotropic hypogonadism with extremely low testosterone concentrations and elevated gonadotropin levels could be diagnosed before gonadectomy. However, a compensated hypergonadotropic hypogonadism with increased gonadotropin levels and still normal testosterone levels was found in all of the other patients with available data (patients 1–6) during the observational period.

Serum concentrations of FSH were highly elevated during the whole course of puberty and increased over time in eight patients with available data (patients 1–6 and 8–9, Fig. 3). In accordance to this, the Sertoli cell markers AMH and inhibin B were below the reference ranges in nine patients (patients 1–6 and 8–10, Figs. 4 and 5), irrespective of the presence of Müllerian structures.

Laboratory assessment of adrenal function of patients 1–6 and 9–10 did not indicate primary adrenal insufficiency. In patient 7 and 8, no data regarding the adrenal function were available. In none of the patients hydrocortisone replacement therapy was reported during the observational period.

### Sex assignment

Patient 1 grew up as male entirely. Patients 2–4 were first assigned female after birth but reassigned as male due to a 46,XY karyotype and further evaluation during early infancy. Patient 6 was raised as female until the age of 9.6 years when increasing signs of virilization occurred and then chose to live in the male gender. Patients 5 and 7–10 grew up as females and did not change sex assignment during evaluation period. Overall, at time of puberty, four patients lived as males (patients 1–4), one patient changed from female to male gender (patient 6), and five patients lived as females (patients 5 and 7–10). In patient 8, psychological evaluation confirmed female gender identity [23]. The other patients did not undergo professional evaluation of gender identity but expressed their favored assigned gender during medical follow-up examinations.

### Treatment

Patients 3 and 4 received testosterone treatment during the observational period starting at the age of around 15 years due to low testosterone levels (patient 3) or a hypoplastic phallus (patient 4). Patients 5, 8, and 10 received GnRH analogue for suppression of pubertal development at a median age of 14.3 years (range 13.1–14.9 years). Estradiol therapy was started in patients 5 and 7–10 at a median age of 15.4 years (range 14.3–16 years) for induction of feminization. Patients 7 and 10 additionally received gestagen therapy at the age of 29 years (patient 7) or 15.6 years (patient 10). Four patients with ambiguous genitalia at birth underwent several surgical procedures to reconstruct male genital appearance during infancy (patient 1 and 3–4) or at early puberty (patient 6). In patients 7–10, gonads were removed at a median age of 17.4 years (range 15.3–17.8 years). In patients 8 and 10, histology revealed testicular tissue with Sertoli-cell-only pattern as well as parts of epididymis and ductus deferens. In patient 9, rudimentary testicular tissue with fibrosis, some ducts and Leydig cells could be detected. No patient showed signs of gonadal malignancy. Gonadal histology of patient 7 was not available.

### Genetic diagnosis

Genetic diagnosis via detection of a *NR5A1* mutation was made at a median age of 15.0 years (range 12.2–31.5 years). In our cohort, three missense mutations (p.T40P, p.V369F, and p.L230R), five frameshift mutations (p.Y211TfsX83, p.L105EfsX45, p.L401AfsX2, p.Q460KfsX42, and p.A176HfsX22), and one in-frame deletion (p.P221_L230del) could be detected via sequencing of the *NR5A1* gene (Table 1). Functional analysis of mutations p.Y211TfsX83, p.T40P, and p.L230R revealed a deleterious impact on transactivation activity on the *AMH* and *STAR* promotor as well as for p.T40P and p.L230R a severe reduction of the DNA binding capacity [23, 24]. Prediction algorithms like PolyPhen-2, Mutation Taster, and SIFT consider the missense mutations p.T40P, p.L230R, and p.V369F as probably damaging, damaging, or disease-causing. In silico analysis via Mutation Taster of the in-frame deletion p.P221_L230del revealed this mutation as disease-causing. It extends between two helices within the activation function-1. Therefore, it is assumed to lead to a significant change of the protein structure resulting in a reduction of the protein functionality. All frameshift mutations are naturally considered to be deleterious due to the shift of the reading frame.

## Discussion

In this study, we evaluate the pubertal development in a cohort of 10 patients with 46,XY DSD due to *NR5A1* mutations with detailed longitudinal clinical and hormonal data. All patients of our cohort who first presented during infancy showed signs of spontaneous pubertal development and virilization with growth of genitalia and development of genital hair at pubertal age consistent with previously published single case reports [34–36]. Spontaneous signs of virilization at the age of puberty also occurred in all but one patient with until then apparently female external genitalia. Of particular note, increasing virilization at the age of puberty led to the change of gender from female to male in one individual.

Despite serum concentrations of testosterone within the male reference range and spontaneous progression of Tanner stages, all patients living as males at the time of puberty showed impaired testicular growth throughout puberty. This observation is in alignment with previous case reports about 46,XY DSD patients with *NR5A1* mutations, in whom low testicular volume, but normal testosterone concentrations have been reported [35–37]. Therefore, in this patient group a decreased testicular volume does not exclude spontaneous pubertal development and testicular volume does not correlate well with a possibly preserved Leydig cell function in puberty. This has also been reported in other DSD conditions like e.g., Klinefelter syndrome in that testicular volume has been shown to be largely dependent on seminiferous tubule volume and not on Leydig cell compartment [38, 39]. Interestingly, in patients of our cohort who showed rather early pubertal development, testicular volume was within the normal range in early stages of puberty but below the norm at a higher age. In two patients, the testicular volume even decreased during puberty. This leads to the assumption that testes might develop normally up to a certain time but are likely to stop increasing size afterwards.

Moreover, the absence of malignant histologic changes in the gonadectomy specimens in our study deserves attention. This is in line with most published case reports of patients with *NR5A1* mutation and without histologic signs of malignancy. Only one case of germ cell neoplasia in situ in a 13-year-old patient with *NR5A1* mutation has been reported [40]. The tumor risk up to pubertal age in this patient group seems not to be increased but this conclusion is limited due to the small number of histologic data in this study. Therefore, gonads need to be closely monitored. Follow-up studies of larger patient cohorts are needed to determine the prevalence and risk factors of gonadal malignancy in patients with *NR5A1* mutations.

Furthermore, we showed that a significant proportion of 46,XY DSD patients due to *NR5A1* mutations did not manifest with ambiguous genitalia during infancy. On the contrary, they were first evaluated at the time of puberty because of signs of virilization in individuals with female appearance and/or pubertal delay with absent thelarche and/or menarche. Therefore, *NR5A1* mutations should not only be taken into consideration in infants with ambiguous genitalia but need to be considered in individuals with female appearance and signs of virilization, delayed thelarche and/or menarche at pubertal age as well. Interestingly, one individual in our cohort showed normal breast development. This again underlines the broad phenotypic spectrum of 46,XY DSD due to *NR5A1* mutations.

All patients who presented in early infancy had testosterone concentrations within the male reference range during puberty. Surprisingly, in three of these patients, testosterone levels were even above the male reference range during early puberty/adolescence (from the age of 9.7 to 11.0 years) which seems to be related to early pubertal virilization. Only in one patient, testosterone concentrations declined during puberty below the male reference range. Several patients with ambiguous genitalia at birth and male sex assignment who showed spontaneous puberty and normal testosterone values have been reported recently [34–36, 41] supporting the idea of a well preserved Leydig cell function later in life. Interestingly, despite normal testosterone levels, LH levels were elevated in the vast majority of patients and further increased during the course of puberty. An explanation for this could be that Leydig cells have a relevant preserved function but higher LH levels are needed to maintain enough testosterone, possibly because of impaired SF-1 stimulation activity or partial gonadal dysgenesis due to the *NR5A1* mutations. Since SF-1 is differently expressed in fetal and adult Leydig cells, it has been postulated that it plays a more important role for testosterone production in fetal Leydig cells [40]. Testosterone production in adult Leydig cells depends more on LH stimulation [42], consistent with our observation of high testosterone and LH levels during puberty. However, to our knowledge, this is the first report of increased testosterone levels in patients with *NR5A1* mutations leading even to early pubertal development. Hence, mutations in the *NR5A1* gene should not only be considered in patients with normal or low testosterone levels, but also in patients with elevated testosterone levels, in combination with increased or rarely normal LH levels.

Serum concentrations of testosterone before gonadectomy in patients who first presented at time of puberty were highly variable ranging from extremely low levels to levels within the male reference range. Testosterone levels in these patients did not reliably predict the development of clitoral growth, development of pubic/axillary hair or breast development during puberty. To date, only a few case reports of patients with apparently female external genitalia at birth but pubertal signs of virilization and normal testosterone values at the age of puberty have been published [36, 37, 43]. Our description of several 46,XY patients with *NR5A1* mutation and female appearance at birth who fulfill these criteria suggests that this occurs more often than previously assumed.

Whereas, a compensated hypergonadotropic hypogonadism with elevated gonadotropin levels but still normal testosterone levels as recently reported by Faienza et al., was found in all patients with available laboratory data, an evident hypergonaodtropic hypogpnadism with decreased testosterone levels was only observed in two patients with female sex assignment [44].

Additionally, our data demonstrate that pubertal development does not strongly correlate to the degree of virilization of the external genitalia at birth in 46,XY DSD caused by *NR5A1* mutations. All patients who presented in early infancy had very low External Genitalia Scores ranging from 3.5 to 5.5 (EGS in male babies (0–1 months, > 37th weeks of gestation, birth weight 2500–4000 g) with typical genital phenotypes: median = 12, 10th percentile = 10.5, and 90th percentile = 12 [33]). Therefore, several patients were first assigned female after birth but soon reassigned as male due to further evaluation. Interestingly, all EGS were even below the average scores of newborns with 46,XY DSD (median = 8.5, 10th percentile = 5.5, and 90th percentile = 11.5) and rather fell into the range of children with 46,XX DSD (median = 6, 10th percentile = 2.7, and 90th percentile = 9) [33]. Our data suggest that patients with *NR5A1* mutations are even more undervirilized at birth than other 46,XY DSD patients. To date, there are no studies about EGS in patients with *NR5A1* mutations. As all patients of our cohort with ambiguous genitalia at birth spontaneously developed signs of virilization at age of puberty and in part even showed unimpaired male puberty despite a low EGS, this score does not seem to be a good predictor of pubertal development in 46,XY patients with *NR5A1* mutations. Further studies with more patients are needed to investigate the importance of the EGS as a predictive clinical tool for later development of patients with *NR5A1* mutations.

Interestingly, in all patients, serum concentrations of FSH were highly elevated during the whole course of puberty and further increased over time, independent from the presence of Müllerian remnants. In accordance to this, serum concentrations of the Sertoli cell markers inhibin B and AMH were below the male reference ranges in all patients regardless of appearance of external genitalia and gender assignment. However, since most of our patients did not have Müllerian structures detected, a sufficient AMH production by Sertoli cells during embryonal sex development in these patients might be assumed. These observations support the previously postulated idea of a progressive Sertoli cell failure over time [37, 41], which seems to be independent of the presence of Müllerian structures. This aspect together with the aforementioned decrease in testicular volume over the course of puberty starting from a normal size, and the fact that *NR5A1* mutations are a known cause for infertility [45–47] highlights the importance to keep the possibility of early spermiogram and cryopreservation in patients with an *NR5A1* mutation in mind.

Remarkably, in all patients who presented in early infancy Müllerian structures were absent. In contrast, in three of five patients with mostly female appearance, Müllerian structures could be detected. This observation could lead to the assumption that Müllerian remnants are more common in less virilized patients due to a global gonadal dysfunction. However, Müllerian structures have also been described in only slightly undervirilized patients in recent case reports [19, 48, 49], and patients with external female genitalia do not always show Müllerian remnants [37]. Therefore, the presence of Müllerian structures is not a good predictor neither for potential virilization at pubertal age nor for the favored gender later in life.

## Conclusion

In conclusion, we demonstrate the broad spectrum of pubertal courses in 46,XY patients with *NR5A1* mutations ranging from spontaneous pubertal progression and virilization despite impaired testicular growth in patients with ambiguous genitalia to signs of virilization and/or pubertal delay with absent thelarche and/or menarche in patients with female appearance. Pubertal development was accompanied by endogenous testosterone production in most patients despite significantly increased gonadotropin levels. The degree of virilization at birth as well as the presence of Müllerian structures did not correlate with the extent of virilization and/or testosterone production during puberty. As development of pubertal changes and also gender identity are not reliably predictable on the basis of the phenotype at birth, it is important to consider sex assignment carefully and to avoid irreversible procedures during infancy.
